# CDK9 inhibition induces epigenetic reprogramming revealing strategies to circumvent resistance in lymphoma

**DOI:** 10.1186/s12943-023-01762-6

**Published:** 2023-03-30

**Authors:** Elana Thieme, Nur Bruss, Duanchen Sun, Edward C. Dominguez, Daniel Coleman, Tingting Liu, Carly Roleder, Melissa Martinez, Krystine Garcia-Mansfield, Brian Ball, Patrick Pirrotte, Lili Wang, Zheng Xia, Alexey V. Danilov

**Affiliations:** 1 City of Hope National Medical Center, 1500 E Duarte Road, Duarte, CA 91010 USA; 2 Knight Cancer Institute, Oregon Health & Science University, Portland, OR USA; 3 Division of Bioinformatics and Computational Biology, Department of Medical Informatics and Clinical Epidemiology, Oregon Health & Science University, Portland, OR USA; 4 Present address: School of Mathematics, Shandong University, Jinan, 250100 Shandong China; 5 Translational Genomics Research Institute, Phoenix, AZ 85004 USA; 6 Integrated Mass Spectrometry Shared Resource, City of Hope National Medical Center, Duarte, CA USA; 7 Biomedical Engineering Department, Oregon Health & Science University, Portland, OR USA

**Keywords:** CDK9, BRD4, Mediator, Super-enhancer, PI3K

## Abstract

Diffuse large B-cell lymphoma (DLBCL) exhibits significant genetic heterogeneity which contributes to drug resistance, necessitating development of novel therapeutic approaches. Pharmacological inhibitors of cyclin-dependent kinases (CDK) demonstrated pre-clinical activity in DLBCL, however many stalled in clinical development. Here we show that AZD4573, a selective inhibitor of CDK9, restricted growth of DLBCL cells. CDK9 inhibition (CDK9i) resulted in rapid changes in the transcriptome and proteome, with downmodulation of multiple oncoproteins (eg, MYC, Mcl-1, JunB, PIM3) and deregulation of phosphoinotiside-3 kinase (PI3K) and senescence pathways. Following initial transcriptional repression due to RNAPII pausing, we observed transcriptional recovery of several oncogenes, including *MYC* and *PIM3*. ATAC-Seq and ChIP-Seq experiments revealed that CDK9i induced epigenetic remodeling with bi-directional changes in chromatin accessibility, suppressed promoter activation and led to sustained reprograming of the super-enhancer landscape. A CRISPR library screen suggested that SE-associated genes in the Mediator complex, as well as *AKT1*, confer resistance to CDK9i. Consistent with this, sgRNA-mediated knockout of *MED12* sensitized cells to CDK9i. Informed by our mechanistic findings, we combined AZD4573 with either PIM kinase or PI3K inhibitors. Both combinations decreased proliferation and induced apoptosis in DLBCL and primary lymphoma cells in vitro as well as resulted in delayed tumor progression and extended survival of mice xenografted with DLBCL *in vivo*. Thus, CDK9i induces reprogramming of the epigenetic landscape, and super-enhancer driven recovery of select oncogenes may contribute to resistance to CDK9i. PIM and PI3K represent potential targets to circumvent resistance to CDK9i in the heterogeneous landscape of DLBCL.

**Supplementary Information:**

The online version contains supplementary material available at 10.1186/s12943-023-01762-6.

## Introduction

The cyclin-dependent kinase (CDK) family of proteins plays an integral role in the regulation of cell cycle progression (e.g., CDK1/2/4/6) and RNA transcription (e.g., CDK7/8/9/11). Deregulated expression of CDKs due to the translocation of chromosomal regions or gene amplifications has been implicated in oncogenesis, rendering CDKs attractive therapeutic targets [1]. Pan-CDK inhibitors (e.g., dinaciclib and flavopiridol) have stalled in development in part due to a narrow therapeutic index. Meanwhile, mounting evidence implicates CDK9, a transcriptional CDK, as a key driver of high-turnover oncogenes, particularly in MYC-dependent cancers [2, 3].

Transcription is tightly regulated at two stages including recruitment of transcriptional machinery to the promoter, and release of RNA polymerase II (RNAPII) from promoter proximal pause. During the pre-initiation complex formation, transcription factors bound to enhancers are looped near the promoter, followed by recruitment of the transcriptional machinery. RNAPII remains paused until BRD4, a preferred cofactor of the mediator complex, recruits CDK9 [4]. Productive elongation of nascent RNA is enabled via phosphorylation of the RNAPII c-terminal domain carried out by CDK7 (Ser5 and Ser7) which releases paused RNAPII and then CDK9 (Ser2) which consequently allows elongation [5]. We have demonstrated that AZ5576, a selective CDK9-inhibitor, led to rapid downmodulation of MYC and Mcl-1 in diffuse large B-cell lymphoma (DBLCL), accompanied by a shutdown of the MYC transcriptional program and apoptosis [2]. Cells expressing high MYC levels were particularly sensitive to CDK9i. AZD4573, a clinical congener of AZ5576, was shown to suppress transcription of the pro-survival proteins Mcl-1 and Bfl-1/A1 in lymphoid tumors, and exhibited anti-tumor effects in AML models *in vivo* via an Mcl-1 dependent mechanism [6, 7]. AZD4573 has entered early-stage clinical trials as monotherapy or in combination for patients with relapse or refractory hematological malignancies (NCT03263637, NCT04630756, NCT05140382). However, limited data explores potential mechanisms of resistance to CDK9i. Here, we investigate cellular effects of CDK9i as well as identify genes and pathways that modulate sensitivity to AZD4573 in DLBCL, an aggressive non-Hodgkin lymphoma (NHL).

## Methods

### Cell culture and primary samples

VAL, OCI-LY3, U-2932, and OCI-LY19 were purchased from DSMZ (Braunschweig, Germany). NU-DUL-1, SU-DHL-4, SU-DHL-6, SU-DHL-10, and SU-DHL-16 were purchased from American Type Culture Collection (atcc.org). Cell lines were maintained in RPMI-1640 medium supplemented with 10% fetal bovine serum (FBS). L4.5, a CD40L-expressing stromal cell line, was purchased from DSMZ and was cultured in Dulbecco’s Modified Eagle Medium (DMEM) 1640 with 10% FBS. Mycoplasma testing was conducted every 3 months using the Mycoplasma PCR Detection Kit (Abcam).

Following approval by the Institutional Review Board and provision of written consent, peripheral blood was obtained from mantle cell lymphoma (MCL; and aggressive NHL subtype) patients at City of Hope Medical Center, with the assistance of the Hematopoietic Tissue Bank. Ficoll-Hypaque technique (Amersham) was used to isolate peripheral blood mononuclear cells (PBMCs). Red blood cells were lysed using ACK lysing buffer (Life Technologies). Primary cells were maintained in RPMI-1640 medium supplemented with 15% FBS, 1% L-glutamine, 1% NEAA and 2% HEPES. All media was supplemented with 1% penicillin–streptomycin (Gibco).

Primary cells were cultured with stroma as described previously [2]. Briefly, primary cells were plated on a layer of stroma at a 50:1 ratio. Cells co-cultured for 24 h then received treatment with experimental compounds. At the end of the experiment, primary cells were gently washed from the stromal layer, transferred into a fresh well for 1 h to minimize sample contamination with residual stromal cells and analyzed.

### Immunoblotting

The following buffer was used to obtain whole cell lysates: Tris–HCL pH 7.4 (20 mM), NaCl (150 mM), EDTA (1 mM), EGTA (1 mM), Na3PO4 (2.5 mM), NaF (5 mM), Triton X-100 (1%), and glycerol (10%), supplemented fresh daily with a phosphatase inhibitor cocktail, 1% PMSF (Sigma Aldrich) and protease inhibitor cocktail (Roche). The following antibodies were used for Western blotting and/or Co-IP: MYC, Mcl-1, Pim-3, JunB, MDM2, pRBP1^S2^, total RBP1, pRB^T821^, total RB, BRD4, MED12, MED26, BAX, AKT, pAKT^T308^, GAPDH, β-Actin, β-tubulin, and horseradish peroxidase conjugated anti-rabbit and anti-mouse antibodies, as well as pRBP1^S5^ and MED14 (Supplemental Table 1).

### Cell viability assays and experimental compounds

Proliferation was quantified using a colorimetric tetrazolium-based assay. Cells were seeded in a 96 well plate at 5000/well and treated with experimental compounds for 72 h. CellTiter96 AQ_ueous_ One reagent (Promega) was added and the optical density was measured at 490 nm after 4 h. IC_50_ was calculated using nonlinear regression with a variable slope.

Apoptosis was quantified as described previously using the ApoScreen Annexin V Apoptosis Kit [2]. Briefly, cells were suspended in Annexin V binding buffer containing 1 µL of Annexin V and 1 µL 7-aminoactinomycin D (7-AAD) per 100 µL of binding buffer. To identify the B-cell population in primary samples, CD19 mAbs (Southern Biotech) was added at the same dilution. After flow cytometry analysis on a LSRFortessa™ (BD Biosciences), data were analyzed with FlowJo software (Tree Star).

AZD4573 was purchased from ChemieTek; JQ1, AZD8835 and copanlisib were from MedChemExpress; AZD1208 and SGI1776 were from Selleckchem.

### Quantitative PCR

Total RNA was extracted using Homogenizer Mini Columns and the total RNA Kit I (Omega Bio-Tek). Complementary DNA (cDNA) was synthesized using qScript cDNA Supermix (QuantaBio), then was prepared according to the manufacturer’s recommendations using PerfeCTaFastMix II (Quantabio) and gene specific probes. The following TaqMan probes from ThermoFischer Scientific were used: *MYC* (Hs00153408_m1), *MCL1* (Hs01050896_m1), *BCL2L1* (Hs00236329_m1), *IRF8* (Hs01128713_m1), *CXCR4* (Hs00607978_s1), *18s* reference gene (Hs99999901_s1). Quantitative real-time PCR (RT-PCR) was carried out using a QuantStudio 7 Flex machine (Applied Biosystems, Foster City, CA). Expression was normalized to the reference gene. Experiments were carried out in triplicate. The comparative C_t_ method was used for analysis (2^−ΔΔCt^, where ΔΔC_t_ = ΔC_tP_ – ΔC_tK_; P = probe and K = reference sample).

### Co-immunoprecipitation

Whole cell lysates were pre-cleared and incubated at 4 °C overnight with 2 µg of BRD4 antibody or rabbit IgG isotype control. Protein A agarose beads (20 µL, Cell Signaling Technology) were added to lysates and samples were incubated for 1.5 h at 4 °C. Beads were washed 3 times in cell lysis buffer (see Immunoblotting), heated to 95 °C for 5 min and subjected to immunoblotting. 10% source protein was used as input control.

### *In vivo* studies

All animal studies were carried out in accordance with institutional guidelines (IACUC #20006). Six-week-old non-obese diabetic/severe combined immunodeficiency/γCnull mice (NOD.Cg-Prkdc^scid^ Il2rg^tm1Wjl^/SzJ [NSG]; the Jackson Laboratory) were xenografted with OCI-LY3 cells (3 × 10^6^ in 200 µL PBS) via flank injection. When tumor volume reached ~ 100 mm^3^, mice were separated into groups and received treatment with AZD4573 [15 mg/kg; diluted in 2%/30%/68% mix of N,N-dimethylacetamide (DMAc), PEG-400, and 1% (v/v) Tween-80; IP; once weekly], copanlisib [15 mg/kg; diluted in PBS; IP; twice weekly], AZD1208 [30 mg/kg, diluted in 0.5% HPMC/0.1% Tween80; oral gavage; twice weekly], drug combinations, or vehicle control. For each treatment group, *n* = 5 mice (10 tumors). Tumor volume was measured three times per week, and mice were euthanized when tumors reached either 20 mm in diameter or ~ 15% of body weight.

### Statistical analysis

All experiments were carried out with a minimum of 3 biological replicates unless otherwise noted. Statistical analysis was performed using the two tailed Student’s t-test in GraphPad Prism (version 9.1.0). *, *p* < 0.05 and **, *p* < 0.01 throughout the manuscript.

### Data availability statement

The mass spectrometry proteomics data have been deposited to the ProteomeXchange Consortium via the PRIDE partner repository with the dataset identifier PXD035858. The ATAC-seq and ChIP-seq data generated in this study are publicly available in Gene Expression Omnibus (GEO) at GSE198851 and GSE210372, respectively.

## Results

### AZD4573 treatment inhibits cell proliferation and induces apoptosis in DLBCL cell lines

**Fig. 1:**
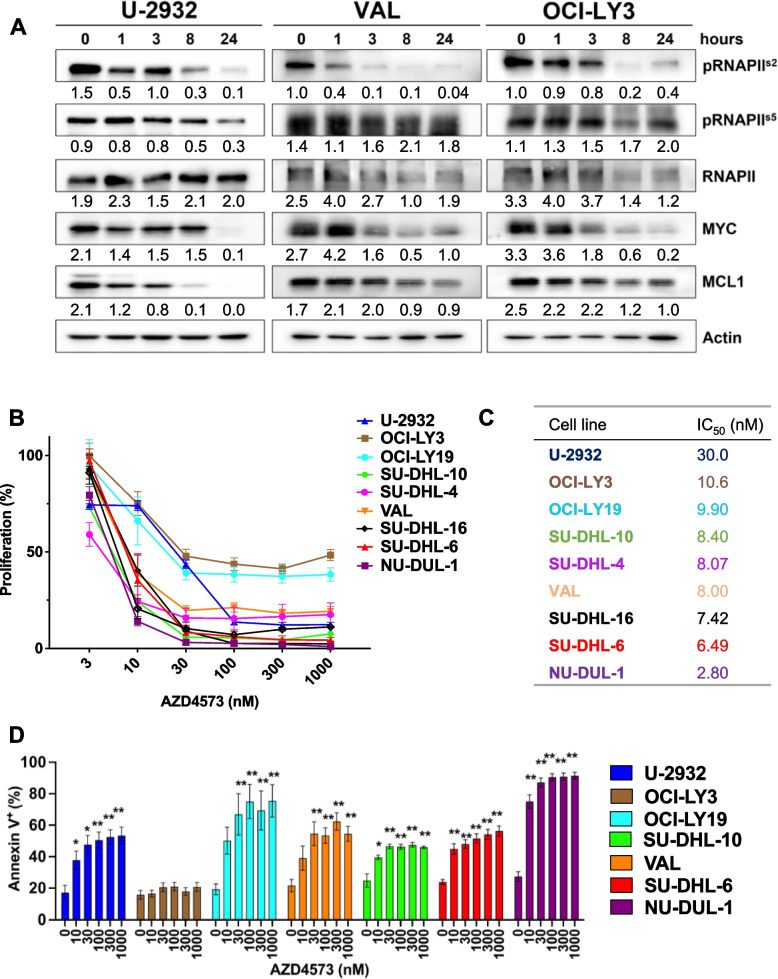
AZD4573 shows preclinical efficacy in DLBCL. **A** Cells were treated with 30 nM AZD4573 as indicated. Whole cell lysates were subjected to immunoblotting. Values for phosphorylated RNAPII are expressed numerically below the blots as a ratio of phosphorylated pRNAPII to total RNAPII, while RNAPII, MYC and MCL1 are expressed as a ratio of protein to Actin. **B-C** Proliferation was assessed in 9 DLBCL cell lines using a colorimetric tetrazolium-based assay, following 48-h treatment. Data is shown as mean ± SEM of three independent experiments, and a table of calculated IC50 values. IC50 was calculated using GraphPad Prism 9 software set to variable slope (four parameters). **D** Apoptosis was tested in 7 DLBCL cell lines treated with AZD4573, measured by flow cytometry at 24 h using Annexin-V staining. Data is shown as mean ± SEM of three independent experiments. *p < 0.05 and **p < 0.01 vs. untreated control

We used the CDK9 inhibitor AZD4573, the clinical congener of AZ5576, as the latter demonstrated pre-clinical efficacy in our earlier studies [2]. In vitro treatment of DLBCL cell lines (U-2932, VAL, OCI-LY3) with AZD4573 led to a time-dependent reduction in phosphorylation of RNAPII^Ser2^ (CDK9 target site) but not at Ser5, consistent with previous reports asserting that AZD4573 selectively inhibits CDK9 (Fig. 1A) [7, 8]. This was accompanied by downmodulation of Mcl-1 and MYC protein levels. AZD4573 potently suppressed proliferation and induced apoptosis in both activated B-cell like (ABC)- and germinal center B-cell like (GCB) DLBCL cell lines (IC_50_ ~ 3–30 nM; Fig. 1B-D). U-2932 and OCI-LY3/LY19 cells were less susceptible to the anti-proliferative effect of CDK9i, and OCI-LY3 cells were resistant to apoptosis, similar to results obtained using AZ5576 [2]. Conversely, the cell lines NU-DUL-1, SU-DHL-6, SU-DHL-16, and VAL showed remarkable sensitivity both via proliferation and apoptosis. AZD4573 appeared more potent than standard chemotherapy drugs in DLBCL cell lines (Supplemental Fig. 1). Thus, CDK9 inhibition with AZD4573 exhibited pre-clinical efficacy in DLBCL cell lines in vitro.

### Oncogene recovery follows CDK9i

**Fig. 2:**
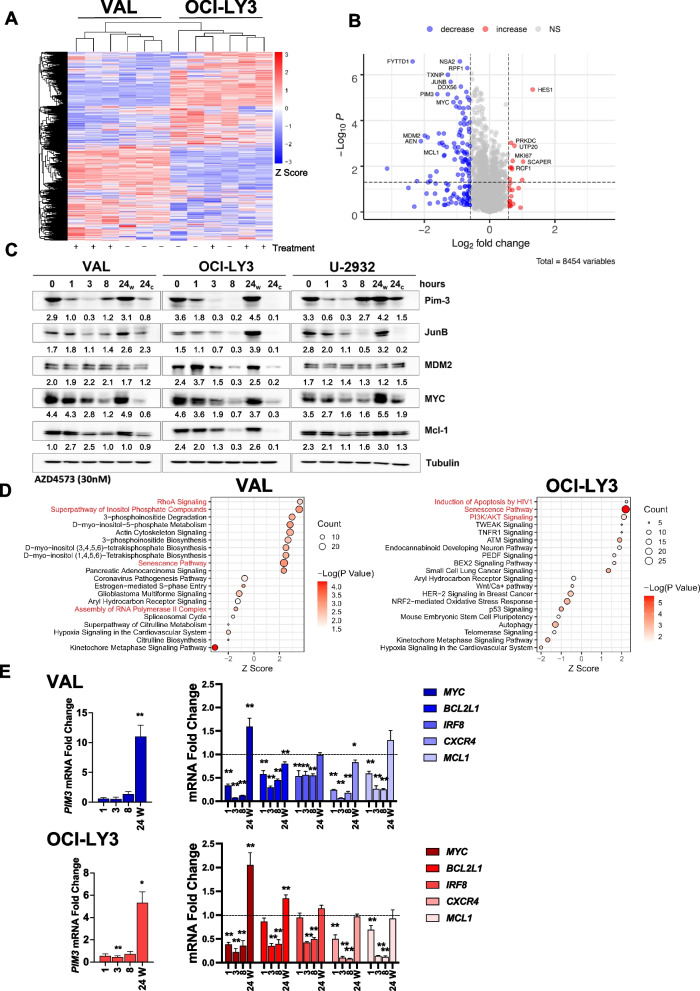
CDK9i transiently suppresses expression of oncoproteins. **A** OCI-LY3 and VAL cells treated with 30 nM AZD4573 or vehicle control for 3 h and subjected to proteomic analysis. Heatmap of all detected proteins is shown. Data are represented as z scores calculated from normalized protein abundance. **B** Volcano plot of all differentially expressed proteins common to both cell lines (|FC| ≥|1.5| treatment versus control; p ≤ 0.05). The identities of select proteins are shown. **C** Cell lines were treated with AZD4573 (30 nM) for 0, 3 and 8 h prior to harvest. After 8 h exposure, the compound was either washed out (w) or not (c = continuous exposure) and harvested after 24 h. Whole cell lysates were subjected to immunoblotting. **D** Top enriched and depleted pathways from IPA analysis of proteomics data from AZD4573-treated VAL and OCI-LY3 cells. Data is presented as a dot plot ranked by pathway Z-score, with size representing the number of genes and color indicating the -Log10 of the significance. **E** VAL (blue) and OCI-LY3 (red) cell were treated with AZD4573 for 0, 3 and 8 h prior to harvest. After 8 h exposure, the compound was washed out and cells were harvested after 24 h. mRNA expression of select genes was quantified by RT-PCR. Data is shown as mRNA fold change in cells treated with AZD4573 versus time-matched cells treated with DMSO. Bars represent mean ± SEM of three independent experiments. Note that plots for the gene *PIM3* were separated from the other genes due to the higher y-axis scale. *p < 0.05 and **p < 0.01, AZD4573 vs. time-matched DMSO control

To understand how AZD4573 impacts key gene expression to contribute to growth inhibition, we characterized changes to protein levels in DLBCL cell lines upon treatment with AZD4573 for 3 h. This early timepoint was chosen to minimize possible off-target effects that may occur at later timepoints. Protein abundance was quantified using liquid chromatography tandem mass spectrometry (LC–MS) in VAL cells, which are highly sensitive to CDK9i, and the relatively resistant OCI-LY3 cells (Fig. 2A; Supplemental Table 2). Data are available via ProteomeXchange with identifier PXD035858.

Seventy-five and one hundred thirteen proteins were differentially expressed following AZD4573 treatment in OCI-LY3 and VAL cells, respectively (|fold change (FC)| ≥|1.5|; p ≤ 0.05). We identified 83 decreased- and 10 increased-abundance proteins common to both cell lines (Fig. 2B). In addition to Mcl-1, a recognized CDK9 inhibitor target, treatment with AZD4573 resulted in decreased abundance of several proto-oncoproteins, including MYC, JunB, and Pim-3. Immunoblotting confirmed rapid downmodulation of these proteins (Fig. 2C). STRING analysis of proteins that were significantly decreased following treatment formed four main functional annotation groups: “Negative regulation of CDK activity”; “Positive regulation of RNA metabolic processes”; “maturation of lsu-rrna” and “Transcription export complex…”, consistent with the expected effect of AZD4573 on RNA transcription (Supplemental Fig. 2A, Supplemental Table 3).

Cells treated with AZD4573 exhibited enrichment of the phosphoinotiside-3 kinase (PI3K) and senescence pathways, including loss of the TP53-degrading E3 ligase MDM2 (Fig. 2D; Supplemental Fig. 2B, Supplemental Table 4). We have previously shown that loss of *TP53* and the pro-apoptotic BH3-only protein *BAX* confers resistance to pharmacologic BH3-mimetics which target Bcl-2 and Mcl-1 in lymphoid and myeloid malignancies [9, 10]. Here we used CRISPR-mediated knockout of *BAX* to demonstrate that its loss similarly conferred partial resistance to AZD4573 (Supplemental Fig. 3). It is likely that *TP53/BAX* network mediates the therapeutic effect of CDK9i due to its well-described effect on Mcl-1.

**Fig. 3:**
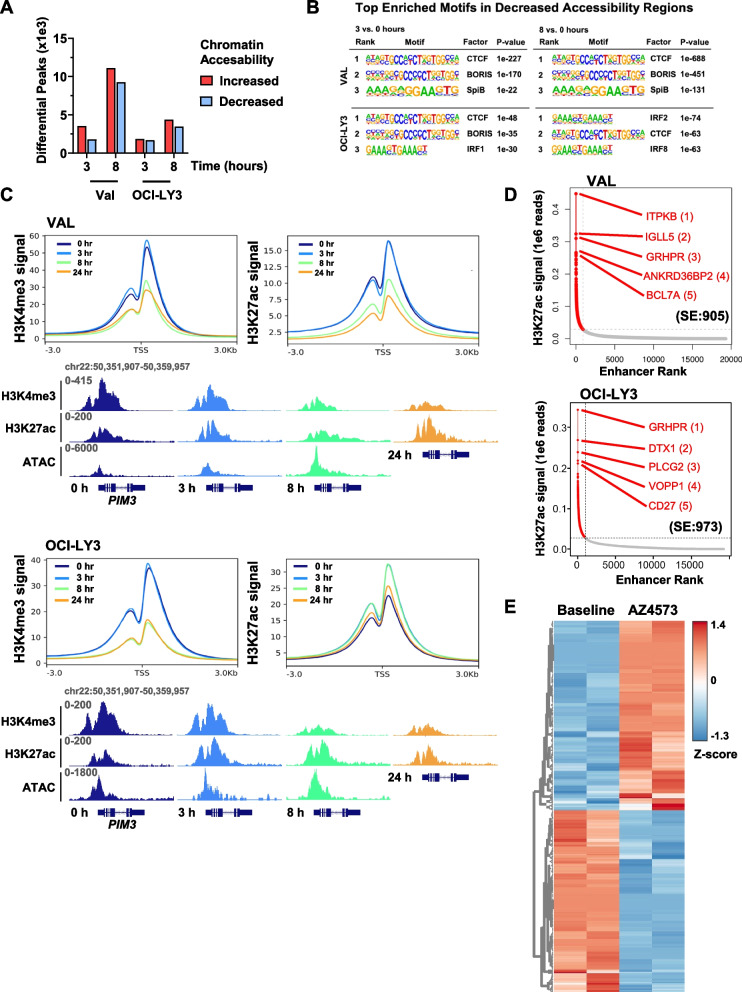
CDK9 inhibition reprograms the promoter and enhancer landscape. OCI-LY3 and VAL cells were treated with AZD4573 (30 nM) for 0, 3 and 8 h prior to harvest. After 8 h exposure, the compound was washed out and cells were harvested after 24 h. Samples were analyzed using ATAC-seq and ChIP-seq. **A** Differential ATAC-seq peaks were calculated using DESeq2 software (|FC| ≥|1.5|; padj ≤ 0.05). Significantly gained and lost peaks are considered regions of increased and decreased chromatin accessibility, respectively. **B** Table of top enriched motifs in regions of decreased chromatin accessibility in ATAC-seq. Table includes position weight matrices of nucleotide sequences comprising motifs identified using gene-based HOMER motif analysis. **C** Metagene analysis of normalized H3K4me3 and H3K27ac ChIP-seq signal intensity plots for all human UCSC genes ± 3 kb of the transcription start site. Gene tracks are shown highlighting the *PIM3* locus. **D** Representative hockey stick plot of super enhancers in Val and OCI-LY3 cell lines. Enhancers were identified and ranked based on H3K27ac ChIP-seq read density as a percentage of total signal, and labeled with the nearest gene. Enhancer ranking was carried out using the ROSE2 algorithm with default parameters. The number of super enhancers per sample is shown in black. SE-associated genes are depicted as red dots. Ranks of 5 top SE-associated oncogenes are included in parenthesis. **E** Heatmap depicting Z-score of genes with differential SEs in VAL cells at 24 versus 0 h of treatment with AZD4573, performed in duplicate

Considering the relatively short half-life of AZD4573 *in vivo*, we treated cells for 8 h followed by washout. Washed cells incubated overnight then were harvested at 24 h from initial treatment. We analyzed the fate of the initially downmodulated proteins over time. Despite the initial downmodulation of MYC, continued exposure to AZD4573 did not result in complete loss of MYC protein levels (Fig. 2C). Furthermore, we observed recovery of Pim3 expression in VAL and U-2932 cells at 8 h despite ongoing CDK9i. Meanwhile, recovery of protein expression occurred by 24 h after washout of the compound (Fig. 2C). To further explore this, we leveraged the previously published RNA-Seq analysis of OCI-LY3 and VAL cells treated with the CDK9-inhibitor AZ5576 [2]. As previously noted, 3-h exposure to 300 nM AZ5576 (IC_50_ in DLBCL cell lines) induced global transcriptional repression, including depletion of *PIM3* and *JUNB* transcripts (Supplemental Fig. 4A). Following the initial transcriptional nadir, certain genes began to exhibit transcriptional recovery despite ongoing CDK9i. We arbitrarily designated “recovery genes” as genes with over 10 counts per million (CPM) at baseline which were downregulated at three hours (Log_2_FC < 0.0) and recovered expression to levels above baseline by 6 h (Log_2_FC > 0.5). A total of 35 and 28 recovery genes were identified in OCI-LY3 and VAL cells, respectively, of which 9 were common to both (Supplemental Fig. 4B). These included genes contributing to lymphomagenesis (*MYC, BCL2L1, IRF8,* and *CXCR4*) along with tumor suppressor genes *CDKN1A* (p21) and the pro-apoptotic BH3-only *BBC3* (PUMA). Of note, *MCL1* did not undergo transcriptional recovery. MYC recovery was also observed by immunoblotting in OCI-LY19 cells treated with AZ5576 as early as at 4 and 6 h, but not in the sensitive SU-DHL-4 or VAL cells (Supplemental Fig. 4C). RNAPII^S2^ phosphorylation was abrogated despite the recovery in MYC protein levels, suggesting that transcriptional recovery was not due to a return of CDK9 function.

**Fig. 4:**
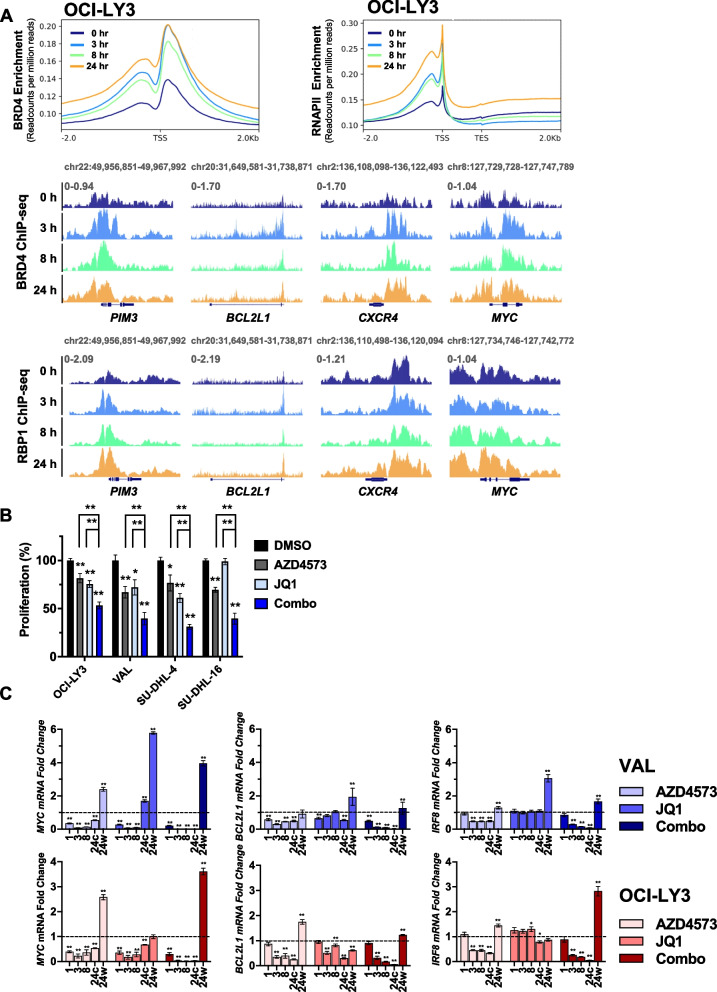
BRD4 enhances transcriptional recovery. **A** OCI-LY3 cells were treated with AZD4573 for 0, 3 and 8 h prior to harvest. After 8 h exposure, the compound was washed off and cells were harvested after 24 h. Samples were analyzed by ChIP-seq for BRD4 and RBP1. Data is shown as normalized ChIP-seq signal intensity plots for all human UCSC genes ± 2 kb, as well as snapshots of select gene tracks. **B** DLBCL cell lines were treated with AZD4573 (3 nM) and the BET-bromodomain inhibitor JQ1 (50 nM) as single agents or in combination for 48 h. Proliferation was analyzed using a colorimetric tetrazolium-based assay. Data is shown as mean ± SEM of three independent experiments. *p < 0.05 and **p < 0.01 vs. untreated control unless otherwise notated. **C** Gene expression fold change of select genes following treatment with AZD4573 at 30 nM, JQ1 at 1 µM, or a combination of the two in VAL and OCI-LY3 cell lines, determined by RT-PCR. Cells were treated for 0, 3 and 8 h prior to harvest. After 8 h exposure, the compound was either washed out (w) or not (c = continuous exposure) and cells were harvested after 24 h. Data is shown as mean ± SEM of three independent experiments. *p < 0.05 and **p < 0.01 vs. time-matched DMSO control

To examine how transcription of these oncogenes behaved under CDK9i with AZD4573, we performed qPCR. AZD4573 potently suppressed transcription of *MYC, BCL2L1, IRF8,* and *CXCR4* as well as *MCL1,* for up to 8 h, while transcript levels of the 18 s housekeeping gene remained stable (Fig. 2E, Supplemental Fig. 4D). Most transcripts recovered following washout, while *MYC* and *BCL2L1* were upregulated 1.35–2 fold above baseline, and *PIM3* was upregulated 5–10 fold (Fig. 2E).

Thus, CDK9i induces rapid changes in the transcriptome and proteome with initial loss of multiple oncogenic transcripts and proteins, followed by transcriptional recovery of certain genes including *MYC* and *PIM3*.

### CDK9i modulates promoter and enhancer architecture

Since epigenetic changes play a significant role in regulation of gene expression, we studied the epigenetic landscape to better understand the mechanisms of transcriptional deregulation following CDK9i. Several factors regulate chromatin accessibility, including posttranslational histone modifications, topological organization of nucleosomes, and positioning of chromatin binding factors. To determine whether changes to the epigenome underly transcriptional deregulation following CDK9i, we first used ATAC-Seq to broadly evaluate the epigenetic landscape following CDK9i. We observed 5,331 and 20,381 differentially accessible regions in VAL cells following 3 and 8 h of AZD4573 treatment, and 3,529 and 7,830 in OCI-LY3 cells, respectively (Fig. 3A, Supplemental Table 5). We observed relatively equal numbers of genomic regions with increased and decreased accessibility. Within regions of decreased chromatin accessibility, we found enrichment of the binding motifs for CCCTC-Binding Factor (CTCF) and its paralogue, CCCTC-Binding Factor Like (BORIS; Fig. 3B, Supplemental Fig. 5A). CTCF establishes the boundaries of topologically associated domains which govern enhancer-promoter communication, and acute CTCF depletion has been shown to alter enhancer-promoter looping [11, 12].

**Fig. 5:**
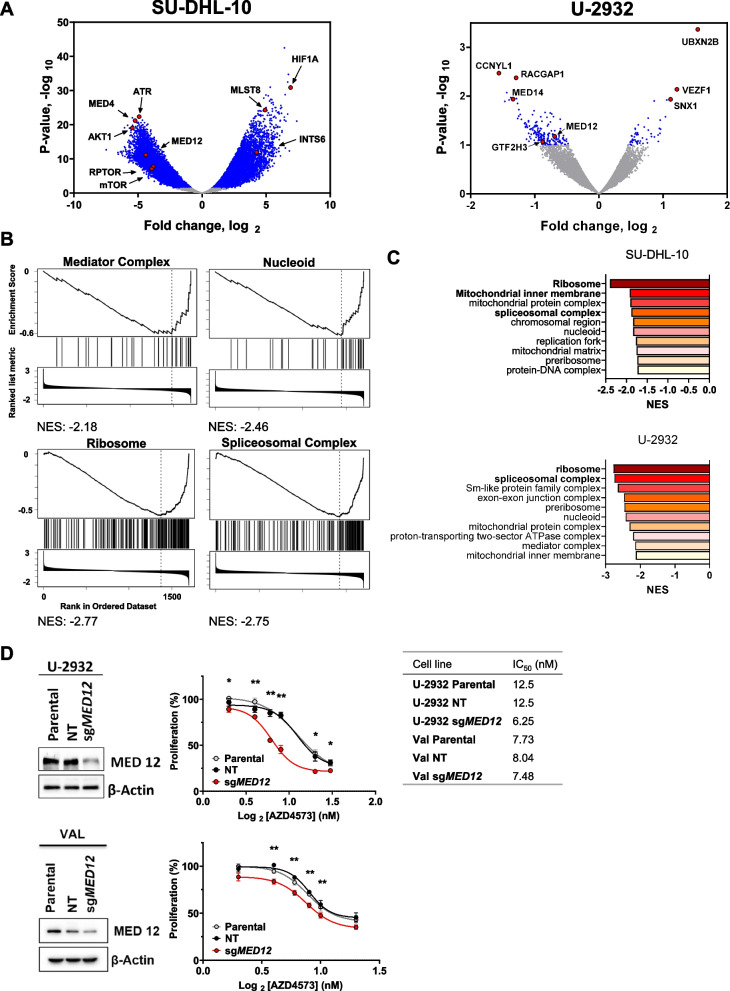
The Mediator complex regulates response to CDK9i. Genome-wide loss of function CRISPR library screening was carried out in U-2932 and SU-DHL-10 cell lines as described in the methods. Data was analyzed using the MaGeCK pipeline. **A** Volcano plot of library screen data in SU-DHL-10 and U-2932 cells. Dots represent the log2(mid fold change) vs. –log10(mid *p*-value) of all sgRNA for one gene in CDK9i-treated cells versus control. Genes with a fold change significance of *p* < 0.1 are depicted in blue and select genes are highlighted in red and identified with a label. **B-C** Gene set enrichment analysis of the library screening was carried out with WebGestalt software. **B** Enrichment plots from U-2932 cells using the Cellular Component gene ontology. **C** Significantly enriched gene sets in SU-DHL-10 and U2932 cells are shown as bar graphs using the Cellular Component gene ontology. **D** *MED12* knockout was established in U-2932 and VAL cells using RNP electroporation as described in the methods. Whole cell lysates were subjected to immunoblotting. Cells were treated with AZD4573 or vehicle control at the indicated concentrations for 48 h. Proliferation was quantified using a colorimetric tetrazolium-based assay. Mean ± SEM is shown. *p < 0.05 and **p < 0.01 vs. NT control. A table of IC50 values is included to the right

Next, we investigated how CDK9i modulates enhancer/promoter architecture. ChIP-Seq for the activating histone marks H3K4me3 and H3K27ac was conducted in DLBCL cells treated with AZD4573 for 8 h, then it was washed out and cells were harvested after 24 h. H3K4me3 is exclusively present at promoters and facilitates binding of RNAPII [13], while H3K27ac is enriched at both enhancers and promoters of actively transcribed genes [14]. While we observed no immediate effect on either marker following short-term treatment with AZD4573 (3 h), H3K4me3 signal enrichment across promoters decreased after 8 h, and this decrease was sustained at 24 h despite washout (Fig. 3C). Promoter H3K27ac signal was similarly lost in VAL cells, but not in OCI-LY3. Despite loss of H3K4me3, epigenetic accessibility at the *PIM3* and *JUNB* promoters increased at 8 h in both tested cell lines, suggesting an alternative promoter activation (Fig. 3C, Supplemental Fig. 5B).

We next characterized the effect of CDK9i on super-enhancer (SE) architecture. SE’s are *cis*-regulatory elements with asymmetrically high enrichment of H3K27ac, BRD4, and the Mediator complex [15, 16]. SE’s regulate diverse genes contributing to lymphoma pathobiology. Enhancers were ranked by H3K27ac signal density and amplitude, followed by assignment of the closest gene. We observed 905 SEs in VAL cells, accounting for 4.7% of all identified enhancers, and 973 SEs in OCI-LY3 cells, 5.4% of identified enhancers (Fig. 3D). Top ranking SE-associated genes included *DTX1* and *PLCG2*, both known to contribute to lymphomagenesis [17, 18].

DLBCL cells exhibited sustained SE reprogramming at the 24 h timepoint (8 h followed by drug washout), with 85 and 20 gained and 92 and 29 lost SEs in VAL and OCI-LY3 cells, respectively (Fig. 3E, Supplemental Table 6). Interestingly, we found that 29% of the previously identified “recovery genes” had SE association in either one or both tested cell lines (e.g. *IRF8, PIM1, BCL2L1, CXCR4*) [19]. Additionally, both cell lines lost a SE proximal to the *PIK3AP1* gene, which encodes a protein that links B-cell receptor signaling with the PI3K/AKT signaling pathway.

In sum, CDK9i induced broad changes to chromatin accessibility, suppressed promoter activation and led to sustained SE reprograming.

### CDK9 inhibition increases binding of BRD4 to chromatin

Transcriptional recovery of *MYC* has been observed by others in HeLa and BJ-TERT cells following treatment with “i-CDK9” or pan-CDK inhibitor flavopiridol and was attributed to a BRD4-dependent mechanism [20, 21]. The BET bromodomain-containing protein BRD4 is a positive regulator of SE-associated genes that binds to hyper-acetylated histones in order to recruit CDK9 [22]. We observed an increase in total BRD4 protein level in DLBCL cells following CDK9i (Supplemental Fig. 6A). To map localization on the genome, ChIP-Seq for BRD4 and RNAPII was carried out. Cells were treated with AZD4573 for 8 h followed by washout. Both BRD4 and RNAPII signals were rapidly enriched at promoter regions following CDK9i, observed as early as 3 h and sustained for up to 24 h despite the washout (Fig. 4A). This enrichment was variable from gene to gene, with *PIM3* showing a more prominent accumulation of RNAPII. Notably, RNAPII exhibited decreased occupancy along gene bodies for the 8 h treatment, indicative of RNAPII promoter proximal pausing. RNAPII enrichment on gene bodies increased following drug washout, suggestive of transcriptional de-repression. Consistent with earlier data generated in HeLa and BJ-TERT cells, this was accompanied by increased complexing between BRD4 and RNAPII (Supplemental Fig. 6B) [20]. Importantly, we found that concurrent continuous targeting of BRD4 and CDK9 significantly attenuated proliferation of DLBCL cells compared to either experimental compound alone (Fig. 4B).

**Fig. 6:**
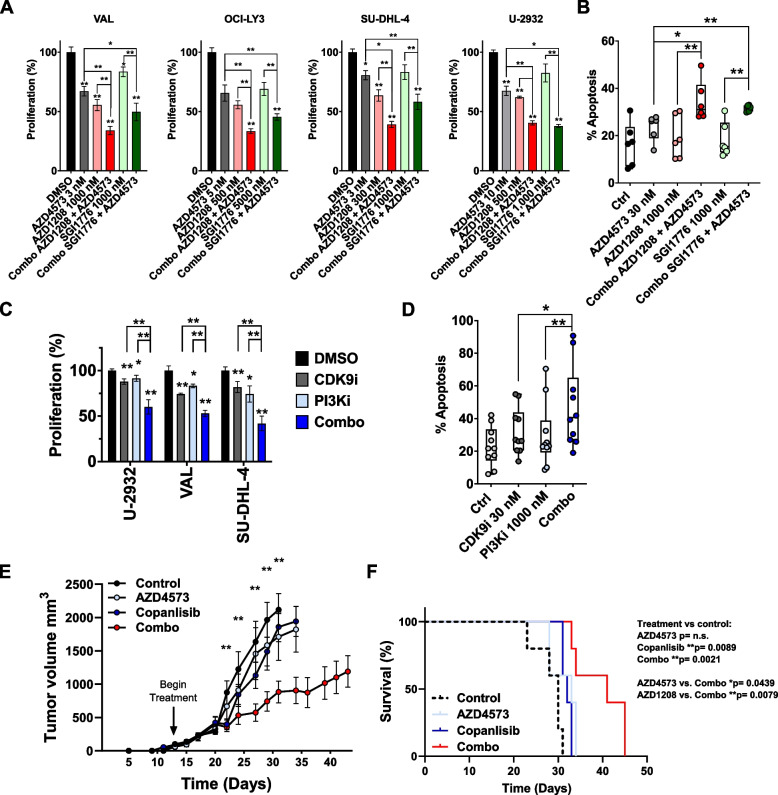
Combination strategies to overcome resistance to CDK9i. **A** DLBCL cell lines were treated with the CDK9 inhibitor AZD4573 and/or the PIM family inhibitor AZD1208, or the PIM1 inhibitor SGI1776, as single agents or in combination at the indicated doses for 48 h. Proliferation was analyzed using a colorimetric tetrazolium-based assay. Data is shown as mean ± SEM of three independent experiments. *p < 0.05 and **p < 0.01 vs. untreated control unless otherwise notated. **B** Primary MCL cells were co-cultured with CD40 ligand expressing stroma for 24 h then were treated with AZD4573, AZD1208, or SGI1776 as single agents or in combination for 48 h. Apoptosis was determined by flow cytometry using Annexin-V-FITC staining. Data is from three patient samples. *p < 0.05 and **p < 0.01. **C** DLBCL cell lines were treated with the CDK9 inhibitor AZD4573 (3 nM) and/or the PI3K inhibitor AZD8835 (100 nM) as single agents or in combination for 48 h. Proliferation was analyzed using a colorimetric tetrazolium-based assay. Data is shown as mean ± SEM of three independent experiments. *p < 0.05 and **p < 0.01 vs. untreated control unless otherwise notated. **D** Primary MCL cells were co-cultured with CD40 ligand expressing stroma for 24 h then were treated with the CDK9 inhibitor AZD4573 or the PI3K inhibitor AZD8835 as single agents or in combination for 48 h. Apoptosis was determined by flow cytometry using Annexin-V-FITC staining. Data is from five patient samples. *p < 0.05 and **p < 0.01. **E–F** Mice were inoculated with OCI-LY3 cells as described in the methods. Once tumor volume reached 100 mm^3^, mice began treatment with AZD4573 (15 mg/kg; IP; once weekly), copanlisib (15 mg/kg; IP; twice weekly), a combination of both, or vehicle control. **E** Tumor growth starting from the first day of engraftment is shown. Data is represented as mean ± SEM of 10 tumors. *p < 0.05 and **p < 0.01, combo treatment versus control. **F** Kaplan–Meier survival curve is shown, significance determined by Log-rank test

Next, we aimed to determine whether BRD4 was necessary for transcription of the “recovery genes”. Concurrent continuous exposure to AZD4573 and BET bromodomain inhibitor JQ1 fully abrogated *MYC*, *BCL2L1,* and *IRF8* mRNA transcription in DLBCL cells (Fig. 4C). However, washout of both compounds resulted in a fourfold upregulation of *MYC* transcription, thus exceeding the degree of *MYC* recovery following AZD4573 alone.

In sum, while inhibition of CDK9 does induce binding of BRD4 to chromatin it is unlikely that BRD4 alone is responsible for the transcriptional recovery of oncogenes, because recovery is still observed when both CDK9 and BRD4 are targeted.

### Disruption of the mediator complex sensitizes cells to CDK9i

We next sought to determine genes and pathways governing the long-term cellular response to CDK9i. To that end, we conducted a genome-wide loss of function CRISPR-cas9 library screening assay. SU-DHL-10 and U-2932 cells were utilized due to their intermediate sensitivity to CDK9i, making them amenable to prolonged exposure to AZD4573. Cas9-expressing cells were transduced with a single guide RNA (sgRNA) library targeting 18,010 genes averaging 5 sgRNAs for every gene. Cells began treatment with 10 nM AZD4573 and the dose was escalated up to 30 nM over the course of 10 days. sgRNAs of the AZD4573-tolerant surviving populations were sequenced. We identified 8,472 depleted and 7,086 enriched sgRNAs in AZD4573-tolerant SU-DHL-10 cells, and 184 and 55 in U-2932 cells, respectively (Fig. 5A, Supplemental Table 7).

*MED4* and *AKT1*, as well as *CCNYL1* and *MED14*, were among the top depleted sgRNAs in SU-DHL-10 and U-2932 cells, respectively, indicating that knockout of these genes sensitized cells to CDK9i (Fig. 5A, Supplemental Fig. 7A). Interestingly, *INTS6* sgRNA was enriched in cells which survived AZD4573, similar to a previously published genome-wide CRISPR-screen which identified that *INTS6* knockout conferred resistance to CDK9i [23]. Enrichment analysis revealed that loss of the ribosome, spliceosome, mitochondrial matrix, nucleoid, and the Mediator complex sensitized DLBCL cells to CDK9i (Fig. 5B-C, Supplemental Fig. 7B; Supplemental Table 8).

Furthermore, *MED12* sgRNA was significantly depleted in both cell lines treated with AZD4573 (Fig. 5A). MED4/12/14 are subunits of the Mediator complex, which is comprised of 4 main modules: the head, middle tail, and the CDK8 kinase module, which regulates the Mediator’s interaction with RNAPII [24]. The Mediator complex localizes to enhancers and links cellular signaling with the recruitment of transcriptional machinery at promoters [24]. We used sgRNA knockout to validate the relevance of Mediator complex subunits to CDK9i susceptibility. While knockout of MED14 and MED26 failed due to technical challenges (data not shown), targeted knockout of the MED12 subunit, a component of the CDK8 kinase module, enhanced cell sensitivity to CDK9i (Fig. 5D). Meanwhile, treatment with AZD4573 did not alter the Mediator complex subunits’ total protein levels (Supplemental Fig. 7C).

Thus, the CRISPR-Cas9 library screen assay suggests that the Mediator complex modulates sensitivity to CDK9i.

### Strategies to overcome resistance to CDK9i

DLBCL has a high degree of inter- and intra-tumor heterogeneity, dictated by factors such as cell of origin and stromal microenvironment, which results in diverse disease biology and clinical course [25]. This guided us to use our findings to investigate the currently targetable genes implicated in our proteomics experiments and the CRISPR-screen to seek out potential combination strategies with AZD4573. Given the recovery of PIM kinase mRNA and protein following CDK9i, we hypothesized that DLBCL cells would be vulnerable to targeting of PIM family kinases in this setting. The PIM family proto-oncogenes encode Ser/Thr kinases upstream of MYC, BCL2L1, and CXCR4 and are commonly overexpressed in B-cell hematologic malignancies, most notably PIM1 [26]. We thus investigated a pan-PIM family kinase inhibition (AZD1208) as well as targeted PIM1 inhibition (SGI1776). Pharmacologic inhibition of PIM kinases (AZD1208 or SGI1776) complemented AZD4573 in a panel of 4 DLBCL cell lines (Fig. 6A). We next tested this combination ex vivo. To mimic the lymph node microenvironment, we used previously established co-cultures of primary MCL cells with CD40L-expressing stroma [9]. The combination of CDK9 and PIM kinase inhibitors induced apoptosis in primary MCL cells to a greater extent than either drug alone (Fig. 6B).

We further tested the anti-tumor efficacy of combined CDK9/PIM1 inhibition *in vivo*. NSG mice were xenografted with OCI-LY3 cells. Once tumors reached 100 mm^3^, mice began treatment with AZD4573 (15 mg/kg; IP; once weekly), the PIM1 inhibitor AZD1208 (30 mg/kg; oral gavage; twice weekly), or a combination of AZD4573/AZD1208. AZD1208 was dosed 24 h after AZD4573, with the aim of suppressing PIM kinase recovery. *In vivo* drug combination slowed DLBCL tumor progression and extended survival compared to control with no apparent toxicity, albeit the difference was not significant compared to either compound alone in this model (Supplemental Fig. 8A-C).

Finally, we sought to investigate *AKT*, which was implicated in resistance to CDK9i in our CRISPR-cas9 library screen. PI3K-AKT is the most frequently activated pathway in human malignancies, and plays a central role in cellular metabolism, survival and proliferation, as well as regulation of histone methylation via EZH2 and KDM5A [27–30]. Combined treatment with AZD4573 and the PI3Kα inhibitor AZD8835 attenuated proliferation of DLBCL cells in vitro to a greater extent than either compound alone (Fig. 6C).

Pro-survival signaling emanating from the stromal microenvironment activates the PI3K-ATK axis and thereby rescues malignant cells from drug-induced apoptosis [31, 32]. Thus, we quantified apoptosis of MCL cells in a stromal co-culture described above. Concurrent targeting of CDK9 and PI3K augmented apoptosis under these conditions (Fig. 6D).

Lastly, we tested the anti-tumor efficacy of combined targeting of CDK9/PI3K *in vivo,* using the mouse model described above. Here mice received treatment with AZD4573 (15 mg/kg; IP; once weekly), the PI3Kαδ inhibitor copanlisib (15 mg/kg; IP; twice weekly), or a combination of both. Copanlisib was dosed 24 h after AZD4573, with the aim of suppressing transcriptional recovery of oncogenes which may be regulated by PI3K. Combined treatment with copanlisib and AZD4573 synergistically slowed tumor growth and extended survival compared to either drug alone (Fig. 6E-F). This combination treatment was not associated with weight loss in mice (Supplemental Fig. 8D).

In sum, informed by our mechanistic findings, here we identified novel strategies to overcome resistance to CDK9i in DLBCL.

## Discussion

Diffuse large B-cell lymphoma (DLBCL) is an aggressive NHL subtype with > 27,000 annual reported cases in the United States alone [33]. Approximately 40% of the patients diagnosed with DLBCL relapse following initial therapy [34]. Furthermore, “double hit” DLBCLs with MYC and BCL2 rearrangement exhibit particularly poor responses to chemotherapy, representing an unmet clinical need. While the emergence of targeted pharmacologic agents inhibiting B-cell receptor associated kinases (e.g., ibrutinib) has revolutionized the therapeutic paradigm in indolent NHLs, responses are modest in DLBCL. This is in part due to a high degree of intra-tumor heterogeneity, which may account for drug-resistance in DLBCL.

CDK9 has been previously established as a target in cancer using genetic and pharmacologic depletion strategies [35–37]. Pharmacologic targeting of CDK9 has shown promising in vitro and *in vivo* anti-tumor activity in malignancies that exhibit dependence on Mcl-1 and/or MYC [3]. Here, we found that CDK9i with the small molecule inhibitor AZD4573 potently thwarted DLBCL survival in vitro. CDK9i downmodulated Mcl-1 and MYC mRNA and proteins levels and led to enrichment of the senescence pathway in DLBCL. The TP53/BAX network mediated sensitivity to CDK9i, likely indicating that the effect of Mcl-1 downregulation still predominates in a fraction of lymphoid cells. Using transcriptomic and proteomic approaches we found that in addition to Mcl-1/MYC, CDK9i resulted in downregulation of multiple other oncogenes, notably Pim-3 and JunB kinases. Given the heterogeneity of lymphoid tumors, this supports a model where multiple factors will be important for sensitivity to CDK9i, not limited to Mcl-1, and will also account for ultimate drug resistance.

Here, we show that CDK9i induces a transcriptional nadir which is followed by transcriptional recovery of certain oncogenes to levels which exceed baseline. Among these oncogenes, MYC and PIM kinases underwent recovery in cells treated with both AZ5576 and its clinical congener AZD4573. To identify potential mechanisms underlying transcriptional recovery, we investigated the effects of CDK9i on the epigenetic landscape. To begin, chromatin accessibility was assessed using ATAC-seq. We found that the CTCF binding motif was highly enriched in genomic regions with decreased accessibility. CTCF is a zinc finger protein which plays an important role in chromatin organization through participation in cis-regulatory elements that regulate the interactions between enhancers and promoters (insulators) [38]. Furthermore, CTCF has been proposed to regulate CDK9 recruitment at the *MYC* locus and has been implicated in cancer-specific transcriptional dysregulation [39, 40]. While further validation is warranted, changes to CTCF binding may play a role in rewriting the epigenetic landscape following CDK9i.

These findings prompted us to further analyze the promoter/enhancer landscape. Interestingly, CDK9i lead to a decrease in promoter activation (H3K4me3), including at recovery gene loci such as *PIM3*. To investigate the enhancer landscape, we focused on regions of the genome with asymmetrically high enrichment of H3K27ac, BRD4, and the Mediator complex, known as super enhancers (SEs), which play an integral role in the maintenance of oncogenic transcriptional programs [16, 41]. Ott et al. found that targeting SEs with BET inhibitors downmodulates oncogenic transcriptional programs in chronic lymphocytic leukemia [19]. While we observed that CDK9i led to loss of enhancers proximal to genes known to contribute to lymphomagenesis, it remains unclear whether the SE reprogramming lends a more indolent or aggressive phenotype to the cell population overall. In fact, our results suggest that concurrent disruption of SE-driven transcription by BET inhibition may exert a synergistic anti-tumor effect when combined with CDK9i.

Prior investigations have similarly reported transcriptional recovery of oncogenes following CDK9i including *JUNB* and *MYC*, attributed to a BRD4-dependent mechanism [20, 21]. We found that CDK9i led to an increase in total BRD4 protein levels, enhanced BRD4 protein-promoter association, as well as RNAPII binding, supporting the hypothesis that BRD4 is involved in maintenance of transcription in the face of CDK9i. Dual BRD4/CDK9 inhibition abrogated oncogene transcription, however *MYC* expression strongly recovered following washout. This suggests that continuous blockade of both CDK9 and BRD4 is required to fully abrogate oncogenic transcription and induce apoptosis, a strategy that will not be feasible in the clinic given the anticipated toxicities of both agents.

We next carried out a genome-wide loss of function CRISPR-cas9 library screening assay and found that knockout of genes in the ribosome, spliceosome, and mediator complex sensitized cells to CDK9i. Given that several subunits of the Mediator complex were highly depleted in the CRISPR screen, we performed a targeted knockout of MED12 and found that loss of MED12 sensitized cells to CDK9i. The Mediator complex cooperates with BRD4 to regulate transcription of SE-associated genes [22, 41–43]. While the Mediator complex serves as both an activator and repressor of transcription, MED12 (part of the CDK8 kinase module) has been proposed to serve a similar function to BRD4 in oncogenic transcription [44, 45]. Recent findings indicate that BET inhibition releases the Mediator from chromatin, therefore it is possible that some of the synergistic effects of CDK9i/BET-bromodomain inhibition are attributable to Mediator eviction [20, 45]. Together, these findings highlight the importance of the SE elements BRD4 and the Mediator complex, in relation to CDK9i resistance, and further suggest that reprogramming of SE landscape following CDK9i ultimately leads to drug resistance.

We sought to build upon our findings to identify candidate targets for drug combination strategies and hypothesized that inhibition of specific recovery genes may enhance the efficacy of CDK9i. We observed upregulation of PIM3 following CDK9i, among other genes. The PIM family of Ser/Thr kinases are commonly overexpressed in lymphoid malignancies and regulate MYC-driven oncogenesis via both transcriptional and post-translational mechanisms [26]. PIM kinases phosphorylate Histone H3 thereby promoting CDK9 recruitment and transcription of MYC, while stabilizing MYC protein via direct phosphorylation on Ser329 and Ser62 residues [26, 46]. PIM inhibition was shown to downregulate the MYC transcriptional program in DLBCL cells, further suggesting that PIM recovery following CDK9i observed in our study may contribute to therapeutic resistance [46]. Indeed, dual targeting of PIM and CDK9 restricted proliferation and induced apoptosis in DLBCL cell lines and in primary MCL cells in our study. Combined treatment with AZD4573 and AZD1208 prolonged survival in the OCI-LY3 xenograft models versus control. This finding may be of clinical relevance to other tumors, such as adenocarcinomas which are known to highly express PIM3, while lymphoid malignancies are more reliant on PIM1 [47].

Oncogenic transcriptional programs are driven by the action of protein networks that link cellular signaling (e.g., PI3K/AKT and MYC pathways), with the assembly and activation of transcriptional machinery (e.g., CDK9, MYC, BRD4, and the Mediator complex). For instance, PI3K/AKT pathway activation induces nuclear translocation and binding of the NFκB subunits RelA/p65 to the κB enhancer, which is facilitated by BRD4 [48, 49]. RelA/p65 recruits the Mediator complex to target genes, and the latter recruits CDK9 [50, 51]. We identified PI3K/AKT pathway inhibition as another candidate for synergism with CDK9i using the CRISPR library screen. Development of PI3K/AKT pathway inhibitors has been a subject of considerable effort given the high frequency of pathway dysregulation in cancer [52]. For example, loss of PTEN expression resulting in aberrant PI3K activation, is observed in 37% of DLBCL cases, and is associated with poor survival [28]. In recent years, multiple PI3K inhibitors have been clinically evaluated for the treatment of hematologic malignancies including copanlisib, idelalisib, and duvelisib. We found that dual CDK9/PI3K inhibition slowed proliferation of DLBCL cell lines and primary cells in vitro as well as restricted tumor growth *in vivo*. Thus, the PI3K/AKT pathway is a promising candidate for combination therapy with CDK9 inhibitors.

The diagnosis of DLBDL represents a broad umbrella of malignancies, varying in cell of origin (GCB vs ABC) and mutational burden, with differing biology and clinical outcomes. Moreover, intra-tumor heterogeneity arising from factors such as the local stromal microenvironment and clonal diversity, renders effective therapeutic management of DLBCL an elusive task [25]. Here we show that BRD4 and the Mediator complex play a mechanistic role in evasion of CDK9i and put forth PIM family kinases and the PI3K/AKT pathway as tractable targets for combination therapy with CDK9 inhibitors. Through these findings, we identify effective drug combination strategies in treatment of DLBCL and potentially other hematological malignancies.

## Conclusions

We show that CDK9 inhibition (CDK9i) rapidly depletes oncogenes in lymphoid cells, followed by inevitable transcriptional recovery. CDK9i induces epigenetic remodeling and reprograms the super-enhancer landscape. Resistance to CDK9i may be circumvented via disruption of super-enhancer associated proteins (BRD4, the Mediator complex), or by targeting PIM and PI3K/ATK pathways. Our findings have implications in clinical development of this class of agents.

## Supplementary Information

**Additional file 1: Supplemental Figure 1.** Anti-lymphoma activity of standard chemotherapy drug. **Supplemental Figure 2.** Proteomic analysis of AZD4573-treated cell lines. **Supplemental Figure 3.** BAX knockout desensitizes DLBCL cells to AZD4573. **Supplemental Figure 4.** Oncogene recovery in cells treated with AZ5576. **Supplemental Figure 5.** CDK9i modulates the epigenetic landscape. **Supplemental Figure 6.** BRD4 increases following CDK9i. **Supplemental Figure 7.** The role of the Mediator complex in CDK9i. **Supplemental Figure 8.** In vivo combination of CDK9i/PIMi.

**Additional file 2.**

**Additional file 3.**

**Additional file 4.**

**Additional file 5.**

**Additional file 6.**

**Additional file 7.**

**Additional file 8.**

## Data Availability

Full results of the ATAC-seq analysis are available at https://www.ncbi.nlm.nih.gov/geo/query/acc.cgi?acc=GSE198851. Full results of the ChIP-seq analysis are available at https://www.ncbi.nlm.nih.gov/geo/query/acc.cgi?acc=GSE210372.

## Abbreviations

7-AAD: 7-Aminoactinomycin D
ABC: Activated B-cell like
CDK: Cyclin-dependent kinase
CDK9i: CDK9 inhibitor
DLBCL: Diffuse large B-cell lymphoma
FBS: Fetal bovine serum
GCB: Germinal center B-cell like
NHL: Non-Hodgkin lymphoma
NSG: NOD.Cg-Prkdc^scid^ Il2rg^tm1Wjl^/SzJ
PBMC: Peripheral blood mononuclear cells
PI3K: Phosphoinotiside-3 kinase
RNAPII: RNA polymerase II
SE: Super-enhancer
