# Supplementary material for: CDK9 inhibition induces epigenetic reprogramming revealing strategies to circumvent resistance in lymphoma
Source: Mol Cancer. 2023 Mar 30;22:64. doi: 10.1186/s12943-023-01762-6 (PMC10061728; doi:10.1186/s12943-023-01762-6)
Supplement: Supplementary file 1 — Additional file 1: Supplemental Figure 1. Anti-lymphoma activity of standard chemotherapy drug. Supplemental Figure 2. Proteomic analysis of AZD4573-treated cell lines. Supplemental Figure 3. BAX knockout desensitizes DLBCL cells to AZD4573. Supplemental Figure 4. Oncogene recovery in cells treated with AZ5576. Supplemental Figure 5. CDK9i modulates the epigenetic landscape. Supplemental Figure 6. BRD4 increases following CDK9i. Supplemental Figure 7. The role of the Mediator complex in CDK9i. Supplemental Figure 8. In vivo combination of CDK9i/PIMi. [file 12943_2023_1762_MOESM1_ESM.docx]

**Supplemental Methods**

*Proteomic Sample Preparation*

Cell pellets were lysed by sonication using an 8M urea lysis buffer in 50 mM Tris-HCl, pH 8.0 containing 1X protease and phosphatase inhibitor cocktail (Pierce). Lysates were clarified by centrifugation at 16,000 g at 4 °C and protein concentration was quantitated using the Pierce BCA Protein Assay Kit (Thermo Scientific). Equal protein amounts (250 µg) were reduced using DTT (5 mM final concentration) for 45 minutes and alkylated using iodoacetamide (10 mM final concentration) for 45 minutes, both at room temperature. Samples were diluted 2-fold prior to a two-hour digestion with rLysC (Wako) at an enzyme to substrate ratio of 1:100. Samples were further diluted 2-fold prior to overnight digestion at 37°C with trypsin (Trypsin Gold, Promega) at an enzyme to substrate ratio of 1:50. Digests were quenched by acidifying samples with formic acid and then desalted using Sep-Pak C18 (Waters) columns. The resulting peptides were vacuum-centrifuged to dryness. For each sample, 90 µg of peptides were labeled using a TMTpro 16-plex kit according to the manufacturer's protocol (Thermo, A44521). Labeled peptides were then pooled, and desalted using Sep-Pak C18 columns. Approximately 500 µg of pooled TMT-labeled peptides were loaded on Waters XBridge C18 column (4.6 x 250 mm, 3.5 µm) and separated by a 96 min gradient using a ternary solvent system A (water), B (acetonitrile) and C (50 mM NH_4_OH, pH 10) operating at a flow rate of 0.5 mL/min. Eluted peptides were collected every 60 seconds on a 96 deep well plate and condensed into 24 fractions for proteomic analysis by pooling every 24th fraction.

*Mass Spectrometry Data Acquisition*

Mass spectrometry data was acquired on Orbitrap Eclipse mass spectrometer (ThermoFisher Scientific) with a FAIMS Pro interface coupled to an Ultimate 3000 RSLCnano liquid chromatograph. Peptides (500 ng peptides from each of the 24 offline fractions) in 5 µL volume of loading solvent (98% water, 2% acetonitrile, 0.1% formic acid) were directly loaded on an EasySpray C18 50cm column (ES903, ThermoFisher Scientific). Using a flowrate of 300 nL/min, peptides were separated over a 120-min gradient formed by Solvent A (Water, 0.1% formic acid) and Solvent B (Acetonitrile, 0.1% formic acid). TMT data were acquired using the TMT-SPS-MS3 workflow with RTS, with 3 FAIMS CV voltages at -40/-55/-70 and a cycle time of 1 s per FAIMS CV [1, 2]. Data Dependent Acquisition (DDA) for MS employed following settings: MS1 scans were acquired in the Orbitrap (resolution= 120K, mass range= 400-1600 m/z, AGC target= 4E5, maximum injection time= 50 ms). Using the top-speed mode, MS2 scans on the most abundant peaks were performed in the iontrap (isolation width= 0.7 Da, CID NCE 30%, AGC target of 100%, max injection time of 35 ms). A dynamic exclusion setting of 45 ms (TMT-SPS-MS3 with RTS) was employed to prevent resampling of same precursors ions. SPS-MS3 scans were performed in the Orbitrap (resolution= 50K, isolation window= 0.7 Da, scan range= 100-500 m/z, maximum injection time= 200 ms, AGC target= 200%, HCD NCE= 55%) on 10 MS2 scan precursors after Real-Time Searching (RTS) of MS2 scans against the embedded Uniprot human proteome with a fixed modification for TMTpro reagent and carbamidomethylation of Cys and dynamic modification for Met oxidation using the RTS search node in Xcalibur 4.4.

*Proteomics data analysis*

Raw spectra were searched against a human protein database (Swissprot/UniprotKB, 2020) using Mascot v2.6 (Matrix Science Ltd) [3] and Proteome Discoverer v2.4 (Thermo Fisher Scientific, Waltham, MA). Tryptic cleavage rules were allowed, with up to two missed cleavages. The precursor mass tolerance was set to 10 ppm, with a fragment mass tolerance of 0.6 Da. Methionine oxidation was set as a variable modification. Carbamidomethyl (C) and TMTpro (K, N-term) were set as fixed modifications. Percolator was used to calculate the false discovery rate (FDR), filtering PSMs and peptides at an FDR of 1%.

Protein copy numbers were computed from raw protein abundances [4] and normalized using internal reference scaling – sample loading (IRS-SL) [5]. A Student’s t-test was used to identify significantly differentially abundant proteins (p-value <0.05, |log_2_(fold-change)| >0.58) between AZD4573 treated and vehicle control samples.

Protein p-values and fold-change direction were used to calculate an H-score (-log_10_(p-value) * ± 1) which was loaded into Gene Set Enrichment Analysis (GSEA) [6] and compared against the Gene Ontology Biological Process knowledgebase (downloaded April 2019) [7]. Biological processes with a GSEA q-value <0.05 were considered significantly enriched.

To identify enriched pathways, significant proteins (p-value <0.05) were loaded into Ingenuity Pathway Analysis (IPA) (QIAGEN Inc., https://www.qiagenbioinformatics.com/products/ingenuity-pathway-analysis) [8] and mapped to the Human Ingenuity Knowledgebase, using log_2_(fold-changes) to calculate pathway z-scores and measure relative activity. Pathways were filtered for a p-value <0.05. Protein interaction analysis was performed using STRING (<https://www.string-db.org>) [9].

The mass spectrometry proteomics data have been deposited to the ProteomeXchange Consortium via the PRIDE partner repository with the dataset identifier PXD035858 [10].

*Genome-wide loss-of-function CRISPR-Cas9 Screen*

Protocol was followed as described previously [11]. Briefly, U-2932 and SU-DHL-10 cells expressing Cas9 were transduced with the human Y. Kosuke sgRNA library targeting 18,010 unique genes, with an average of 5 sgRNA per gene (Addgene, CAT# 67989) [12]. Cell lines were first treated with 10 nM AZD4573 until the dose was escalated up to 30 nM over the course of 10 days. Control cells were treated with DMSO for 10 days. Genomic DNA was harvested, then barcoded sgRNA were amplified and sequenced. Reads were trimmed to expected 19 base length based on presence of expected adaptor sequences using Cutadapt [13]. The trimmed reads were aligned to the sgRNA library using Bowtie2 keeping only uniquely mapped perfect matches as determined by Bamtools [14, 15] . Counts were assigned to the sgRNA database using MaGeCK count pipeline [16]. Statistical analysis was performed using edgeR [17]. Only sgRNAs with ≥100 reads in at least half of the samples were considered for statistical analysis. To calculate the mid P-value, every sgRNA for single gene was ordered by P-value, and the middle sgRNA was selected. Mid fold change was calculated using the same logic. Pathway analysis using GSEA was performed using the signed -log10(p-value) from the mid sgRNA and was carried out using Webgestalt software referencing the Kegg pathway and Gene Ontology databases [11].

*Genome editing by RNP electroporation*

Genome editing by RNP electroporation was executed in VAL and U-2932 cell lines using the Lonza Amaxa Nucleofector II System (Lonza, Basel, Switzerland), 4 μM (1:3, Cas9:sgRNA) Alt-R® (Integrated DNA Technologies, Inc) Cas9 RNP complex and 4 μM Alt-R® Cas9 Electroporation Enhancer (Integrated DNA Technologies, Inc), as described previously [18]. Chemically synthesized guide RNAs (Synthego, CA, USA) were diluted as recommended by the manufacturer. Cas9 protein was added to the guide RNAs then incubated for 10 minutes at room temperature in order to form an RNP complex (IDT). 1.5x10^5^ cells per sample were suspended in PBS (20 μL) and RNP complexes were added (5 μL). Amaxa protocol V-001 was used for electroporation. Experiments were conducted 14 days after electroporation. Target sequences were:

*sgCtrl*: GAGATATCAATCCTCCCGC

*sgMED12*: CCCATACCTATCGGGATCA

*sgBAX*: AGUUUCAUCCAGGAUCGAGC

*ATAC-seq*

Samples were prepared according to the Fast-ATAC protocol, a method optimized for blood cells [19]. Briefly, 5X10^4^ cells were suspended in 22 µL of dPBS, and were added to 28 µL of transposase mixture (25 µL of 2x TD buffer, 2.5 µL of TDE1, 0.5 µL of 1% digitonin) (Illumina; Promega). The samples were incubated at 37°C for 30 minutes in a simpliamp thermocycler. DNA was purified using a DNA Clean and Concentrator-5 capped kit (Zymo). The transposed DNA fragments were amplified for single index sequencing using custom Nextera PCR primers 1 and 2 as previously described [20].

For ATAC-seq analysis, we followed the ATAC-seq guidelines published on Harvard FAS informatics (https://informatics.fas.harvard.edu/atac-seq-guidelines.html) to process the raw ATAC-seq fastq files. Briefly, reads were aligned to the hg19 reference genome using bowtie2 [14] with “--very-sensitive” option. After removing mitochondrial reads, non-uniquely aligned reads, and PCR duplicates, MACS2 [21] with parameter “-q 0.05” was used to perform peak calling for each sample. To obtain a consensus-set of peaks across samples, peak files were merged using the ‘merge’ function in bedtools [22]. The number of reads that fell into the merged peaks was obtained using the ‘multicov’ function in bedtools, with parameters “-q 10” to exclude reads with poor mapping quality, and “-f 0.05” to exclude reads that only minimally overlap the peak. The obtained read counts for peaks were processed in DESeq2 [23] to identify hyper- and hypo-accessible peaks across different conditions. Peaks were called hyper or hypo-accessible using the absolute fold change larger than 1.5 and adjusted p-value less than 0.01 from DESeq2. Full results of the ATAC-seq analysis are available at https://www.ncbi.nlm.nih.gov/geo/query/acc.cgi?acc=GSE198851

*ChIP-seq*

Chromatin immunoprecipitations were performed using the SimpleChIP Enzymatic Chromatin IP Kit with Magnetic Beads (Cell Signaling Technology #9003). The following antibodies were used: Rbp1 CTD 4H8 (CAT# 2629, CST), H3K27ac (CAT# 8173, CST), H3K4me3 (CAT# 9751, CST), BRD4 (CAT# A301-985A, Bethyl), Rabbit IgG (CAT# 2729, CST). Sequencing libraries were prepared using the SimpleChIP ChIP-seq DNA Library Prep Kit for Illumina (Cell Signaling Technology #56795) with Dual Index Multiplex Oligos for Illumina (Cell Signaling Technology #47538). Paired-end sequencing was performed on an Illumina HiSeq PE150 instrument. Libraries were sequenced to depths ranging from approximately 25-50 million reads.

The raw FASTQ files were trimmed with Trimmomatic (v0.39) [24] to cut adapter and other Illumina-specific sequences from the read and only keep the reads longer than 36. Then, the trimmed FASTQ data was mapped to the human reference genome hg19 with Bowtie2 v2.2.9 [14] with default settings. Mapped reads were further filtered to remove reads with a mapping quality (q score) less than 10 and duplicated reads using samtools (v1.3.1) [25]. To visualize occupancy, meta plots were generated using bamCompare, computeMatrix and plotProfile within deepTools2 (v3.5.1). Full results of the ChIP-seq analysis are available at https://www.ncbi.nlm.nih.gov/geo/query/acc.cgi?acc=GSE210372

*Super-enhancer (SE) identification*

We conducted the SE identification by using ROSE2 (https://github.com/BradnerLab/pipeline) [26], with the default parameters [27]. First, given the processed BAM file of each sample, MACS2 (v2.2.7.1) [21] was used to call peaks with a q value cutoff of 0.05. Genomic loci comparison of the bam files were performed with BEDOPS (v2.4.35) [28]. Reads mapping to genomic loci and density analysis were performed with Bamliquidator v1.2.0 [26]. Enhancers were identified and ranked based on H3K27ac ChIP-seq read density as a percentage of total signal and labeled with the nearest gene. [26] The target genes of super-enhancers for subsequent analyses were extracted from ROSE2 output file "ENHANCER_TO_TOP_GENE.txt".

*Differential SE analysis*

Differential SE analysis was performed as outlined in Ott *et al.* [27]. First, we used the union of all SEs across samples as regions of interest for the downstream analysis. Then, we employed DESeq2 [23] to estimate within-group variance in sample cohorts controlling for GC content of SE union regions and technical variability in sample read depth within SEs. We used Rsubread Bioconductor R package to count reads in each SE union region. We resolved the multi-overlapping read assignment by the length of overlap, and overlapping regions by using chromVAR [29] 'filterPeaks' function. GC content for each region was calculated by the Biostrings Bioconductor package (https://rdrr.io/bioc/Biostrings/) and offset matrix was generated by EDASeq [30]. The normalization factors were further updated to make geometric means of each row of the offset matrix equal to 1. Finally, DESeq2 was used to compare two conditions to define differential SEs by the Benjamini-Hochberg adjusted p-value <0.05.

**Supplemental Figures**

**Supplemental Figure 1. Anti-lymphoma activity of standard chemotherapy drug.** Proliferation was assessed in DLBCL cell lines using a colorimetric tetrazolium-based assay, following 48-hour drug treatment. Data is shown as mean ± SEM of three independent experiments.

**Supplemental Figure 2. Proteomic analysis of AZD4573-treated cell lines.**

OCI-LY3 and VAL cells treated with 30 nM AZD4573 or vehicle control for 3 hours and subjected to proteomic analysis.

(**A**) Protein-protein interaction (PPI) networks of proteins with decreased abundance. Networks were generated using STRING (|FC| ≥ |1.5| treatment versus control, p ≤ 0.05, in both VAL and OCI-LY3; https://string-db.org/). Nodes represent proteins, color coded by cluster. Edges represent protein-protein association, confidence indicated by thickness. Top network clusters are circled and identified.

**(B**) IPA analysis of proteomics data from AZD4573-treated VAL and OCI-LY3 cells was carried out. Table of select genes in the IPA senescence pathway, with fold change and significance of AZD4573 treatment vs DMSO in VAL and OCI-LY3.

**Supplemental Figure 3. BAX knockout desensitizes DLBCL cells to AZD4573.**

*BAX* knockout was established in OCI-LY3 and VAL cells using RNP electroporation as described in the methods. Whole cell lysates were subjected to immunoblotting. Cells were treated with AZD4573 or vehicle control at the indicated concentrations for 48 hours. Proliferation was quantified using a colorimetric tetrazolium-based assay. Mean±SEM is shown. *p<0.05 and **p<0.01 vs. NT control.

**Supplemental Figure 4. Oncogene recovery in cells treated with AZ5576.**

(**A**) Volcano plots of mRNA fold-change. DLBCL cells were incubated with 300 nM AZ5576 for 0, 3 or 6 hours and were analyzed by RNA-seq. The number of significantly down/upregulated genes is indicated at the top left and right hand corners (|FC| ≥ |1.5| 3 or 6 hours versus 0 hours; p ≤ 0.05). The genes *JUNB* and *PIM3* are depicted in red and pointed out with arrows in the 3 h vs 0 h comparison.

(**B**) Table of recovery genes. DLBCL cells were incubated with 300 nM AZ5576 for 0, 3 or 6 hours and analyzed by RNA-seq. We arbitrarily designated “recovery genes” as genes with over 10 counts per million (CPM) at baseline which were downregulated at three hours (Log_2_FC < 0.0, 3 hours vs baseline) and rebounded expression to levels at or above baseline (Log_2_FC > 0.5, 6 hours vs baseline).

(**C**) Cells were treated with 300 nM AZ5576 as indicated (continuously). Whole cell lysates were subjected to immunoblotting at the indicated timepoints.

(D) VAL (blue) and OCI-LY3 (red) cell were treated with AZD4573 for 0, 3 and 8 hours prior to harvest. After 8-hour exposure, the compound was either washed out (w) or not (c=continuous exposure) and cells were harvested after 24 hours. mRNA expression of the housekeeping gene, 18s, was quantified by RT-PCR. The y-axis shows the quantification cycle (Cq) value for RNA amplification. Bars represent mean ± SEM of three independent experiments. *p<0.05 and **p<0.01, AZD4573 vs. time-matched DMSO control.

**Supplemental Figure 5.** **CDK9i modulates the epigenetic landscape.**

OCI-LY3 and VAL cells were treated with AZD4573 (30 nM) for 0, 3 and 8 hours prior to harvest. After 8-hour exposure, drug was washed out and cells were harvested after 24 hours. Samples were analyzed using ATAC-seq and ChIP-seq.

(**A**) Table includes position weight matrices of nucleotide sequences comprising motifs identified using gene-based HOMER motif analysis.

(**B**) Gene tracks are shown highlighting the *JUNB* locus for H3K4me3 and H3K27ac ChIP-seq and ATAC-seq.

**Supplemental Figure 6. BRD4 increases following CDK9i**

(**A**) OCI-LY3, VAL and SU-DHL-4 cells were incubated with AZD4573 (30 nM). Whole cell lysates were extracted at the indicated timepoints and subjected to immunoblotting.

(**B**) Co-IP of BRD4 from the whole cell lysate of OCI-LY3 cells treated with AZD4573 (30 nM). Immunoprecipitated proteins were immunoblotted for the RNAPII subunit RBP1.

**Supplemental Figure 7. The role of the Mediator complex in CDK9i.**

(**A**) Box and whisker plot of select highly significant gene knockouts from the genome-wide screen in SU-DHL10 and U-2932 cells, with individual sgRNA depicted as points.

(B) Significantly enriched gene sets in SU-DHL-10 and U2932 cells are shown as bar graphs using the KEGG pathway.

(C) Cell lines were treated with AZD4573 (30 nM) for 0, 3 and 8 hours prior to harvest. After 8-hour exposure, drug was either washed out (w) or not (c=continuous exposure) and harvested after 24 hours. Whole cell lysates were subjected to immunoblotting.

**Supplemental Figure 8. In vivo combination of CDK9i/PIMi.**

(**A-C**) Mice were inoculated with OCI-LY3 cells as described in the methods. Once tumor volume reached 100 mm^3^, mice began treatment with AZD4573 (15 mg/kg; IP; once weekly), AZD1208 (30 mg/kg; oral gavage; twice weekly), a combination of both, or vehicle control.

(**A**) Tumor growth starting from the first day of engraftment is shown.

(**B**) Kaplan-Meier survival curve is shown, significance determined by Log-rank test.

(**C**) Body weight starting 2 weeks post engraftment is shown. Data is represented as mean ± SEM of 5 mice. *p<0.05 and **p<0.01, treatment versus control.

(**D**) Mice were inoculated with OCI-LY3 cells as described in the methods. Once tumor volume reached 100 mm^3^, mice began treatment with AZD4573 (15 mg/kg; IP; once weekly), copanlisib (15 mg/kg; IP; twice weekly), a combination of both, or vehicle control. Body weight starting 2 weeks post engraftment is shown. Data is represented as mean ± SEM of 5 mice. *p<0.05 and **p<0.01, treatment versus control.

**Supplemental Table 01**

| Target | Catalogue # | Vendor |
| --- | --- | --- |
| c-Myc | 9402 | Cell Signaling Technology (C ST) |
| Mcl-1 (D2W9E) | 94296 | CST |
| PIM3 | 4165 | CST |
| JUNB | 3753 | CST |
| MDM2 | 86934 | CST |
| P-Rpb1 CTD (S2) (E1Z3G) | 13499 | CST |
| Rpb1 CTD | 2629 | CST |
| pRB (Thr821) | 44-582G | Invitrogen |
| RB | 9313 | CST |
| BRD4 | A301-985A | Bethyl |
| MED12 | 4529 | CST |
| MED26 | 13641 | CST |
| Bax | 5023 | CST |
| AKT | 9272 | CST |
| pAKT t308 | 13038T | CST |
| GAPDH | 5174 | CST |
| beta-Actin (D6A8) | 8457 | CST |
| beta-tubulin | 5568 | CST |
| Rbp1 (S5) | 920304 | Biolegend |
| MED14 | ab72141 | AbCam |

**Supplemental References**

1. Schweppe DK, Eng JK, Yu Q, Bailey D, Rad R, Navarrete-Perea J, Huttlin EL, Erickson BK, Paulo JA, Gygi SP: **Full-Featured, Real-Time Database Searching Platform Enables Fast and Accurate Multiplexed Quantitative Proteomics.** *J Proteome Res* 2020, **19:**2026-2034.

2. Yu Q, Paulo JA, Naverrete-Perea J, McAlister GC, Canterbury JD, Bailey DJ, Robitaille AM, Huguet R, Zabrouskov V, Gygi SP, Schweppe DK: **Benchmarking the Orbitrap Tribrid Eclipse for Next Generation Multiplexed Proteomics.** *Anal Chem* 2020, **92:**6478-6485.

3. Perkins DN, Pappin DJ, Creasy DM, Cottrell JS: **Probability-based protein identification by searching sequence databases using mass spectrometry data.** *Electrophoresis* 1999, **20:**3551-3567.

4. Wisniewski JR, Hein MY, Cox J, Mann M: **A "proteomic ruler" for protein copy number and concentration estimation without spike-in standards.** *Mol Cell Proteomics* 2014, **13:**3497-3506.

5. Plubell DL, Wilmarth PA, Zhao Y, Fenton AM, Minnier J, Reddy AP, Klimek J, Yang X, David LL, Pamir N: **Extended Multiplexing of Tandem Mass Tags (TMT) Labeling Reveals Age and High Fat Diet Specific Proteome Changes in Mouse Epididymal Adipose Tissue.** *Mol Cell Proteomics* 2017, **16:**873-890.

6. Subramanian A, Tamayo P, Mootha VK, Mukherjee S, Ebert BL, Gillette MA, Paulovich A, Pomeroy SL, Golub TR, Lander ES, Mesirov JP: **Gene set enrichment analysis: a knowledge-based approach for interpreting genome-wide expression profiles.** *Proc Natl Acad Sci U S A* 2005, **102:**15545-15550.

7. Ashburner M, Ball CA, Blake JA, Botstein D, Butler H, Cherry JM, Davis AP, Dolinski K, Dwight SS, Eppig JT, et al: **Gene ontology: tool for the unification of biology. The Gene Ontology Consortium.** *Nat Genet* 2000, **25:**25-29.

8. Kramer A, Green J, Pollard J, Jr., Tugendreich S: **Causal analysis approaches in Ingenuity Pathway Analysis.** *Bioinformatics* 2014, **30:**523-530.

9. Szklarczyk D, Gable AL, Lyon D, Junge A, Wyder S, Huerta-Cepas J, Simonovic M, Doncheva NT, Morris JH, Bork P, et al: **STRING v11: protein-protein association networks with increased coverage, supporting functional discovery in genome-wide experimental datasets.** *Nucleic Acids Res* 2019, **47:**D607-D613.

10. Perez-Riverol Y, Bai J, Bandla C, Garcia-Seisdedos D, Hewapathirana S, Kamatchinathan S, Kundu DJ, Prakash A, Frericks-Zipper A, Eisenacher M, et al: **The PRIDE database resources in 2022: a hub for mass spectrometry-based proteomics evidences.** *Nucleic Acids Res* 2022, **50:**D543-D552.

11. Nechiporuk T, Kurtz SE, Nikolova O, Liu T, Jones CL, D'Alessandro A, Culp-Hill R, d'Almeida A, Joshi SK, Rosenberg M, et al: **The TP53 Apoptotic Network Is a Primary Mediator of Resistance to BCL2 Inhibition in AML Cells.** *Cancer Discov* 2019, **9:**910-925.

12. Tzelepis K, Koike-Yusa H, De Braekeleer E, Li Y, Metzakopian E, Dovey OM, Mupo A, Grinkevich V, Li M, Mazan M, et al: **A CRISPR Dropout Screen Identifies Genetic Vulnerabilities and Therapeutic Targets in Acute Myeloid Leukemia.** *Cell Rep* 2016, **17:**1193-1205.

13. Martin M: **Cutadapt removes adapter sequences from high-throughput sequencing reads.** *2011* 2011, **17:**3 %J EMBnet.journal.

14. Langmead B, Salzberg SL: **Fast gapped-read alignment with Bowtie 2.** *Nat Methods* 2012, **9:**357-359.

15. Barnett DW, Garrison EK, Quinlan AR, Strömberg MP, Marth GT: **BamTools: a C++ API and toolkit for analyzing and managing BAM files.** *Bioinformatics* 2011, **27:**1691-1692.

16. Li W, Xu H, Xiao T, Cong L, Love MI, Zhang F, Irizarry RA, Liu JS, Brown M, Liu XS: **MAGeCK enables robust identification of essential genes from genome-scale CRISPR/Cas9 knockout screens.** *Genome Biol* 2014, **15:**554.

17. Dai Z, Sheridan JM, Gearing LJ, Moore DL, Su S, Wormald S, Wilcox S, O'Connor L, Dickins RA, Blewitt ME, Ritchie ME: **edgeR: a versatile tool for the analysis of shRNA-seq and CRISPR-Cas9 genetic screens.** *F1000Res* 2014, **3:**95.

18. Vakulskas CA, Dever DP, Rettig GR, Turk R, Jacobi AM, Collingwood MA, Bode NM, McNeill MS, Yan S, Camarena J, et al: **A high-fidelity Cas9 mutant delivered as a ribonucleoprotein complex enables efficient gene editing in human hematopoietic stem and progenitor cells.** *Nat Med* 2018, **24:**1216-1224.

19. Corces MR, Buenrostro JD, Wu B, Greenside PG, Chan SM, Koenig JL, Snyder MP, Pritchard JK, Kundaje A, Greenleaf WJ, et al: **Lineage-specific and single-cell chromatin accessibility charts human hematopoiesis and leukemia evolution.** *Nat Genet* 2016, **48:**1193-1203.

20. Buenrostro JD, Wu B, Chang HY, Greenleaf WJ: **ATAC-seq: A Method for Assaying Chromatin Accessibility Genome-Wide.** *Curr Protoc Mol Biol* 2015, **109:**21 29 21-21 29 29.

21. Zhang Y, Liu T, Meyer CA, Eeckhoute J, Johnson DS, Bernstein BE, Nusbaum C, Myers RM, Brown M, Li W, Liu XS: **Model-based analysis of ChIP-Seq (MACS).** *Genome Biol* 2008, **9:**R137.

22. Quinlan AR, Hall IM: **BEDTools: a flexible suite of utilities for comparing genomic features.** *Bioinformatics* 2010, **26:**841-842.

23. Love MI, Huber W, Anders S: **Moderated estimation of fold change and dispersion for RNA-seq data with DESeq2.** *Genome Biol* 2014, **15:**550.

24. Bolger AM, Lohse M, Usadel B: **Trimmomatic: a flexible trimmer for Illumina sequence data.** *Bioinformatics* 2014, **30:**2114-2120.

25. Li H, Handsaker B, Wysoker A, Fennell T, Ruan J, Homer N, Marth G, Abecasis G, Durbin R, Genome Project Data Processing S: **The Sequence Alignment/Map format and SAMtools.** *Bioinformatics* 2009, **25:**2078-2079.

26. Lin CY, Erkek S, Tong Y, Yin L, Federation AJ, Zapatka M, Haldipur P, Kawauchi D, Risch T, Warnatz HJ, et al: **Active medulloblastoma enhancers reveal subgroup-specific cellular origins.** *Nature* 2016, **530:**57-62.

27. Ott CJ, Federation AJ, Schwartz LS, Kasar S, Klitgaard JL, Lenci R, Li Q, Lawlor M, Fernandes SM, Souza A, et al: **Enhancer Architecture and Essential Core Regulatory Circuitry of Chronic Lymphocytic Leukemia.** *Cancer Cell* 2018, **34:**982-995 e987.

28. Neph S, Kuehn MS, Reynolds AP, Haugen E, Thurman RE, Johnson AK, Rynes E, Maurano MT, Vierstra J, Thomas S, et al: **BEDOPS: high-performance genomic feature operations.** *Bioinformatics* 2012, **28:**1919-1920.

29. Schep AN, Wu B, Buenrostro JD, Greenleaf WJ: **chromVAR: inferring transcription-factor-associated accessibility from single-cell epigenomic data.** *Nat Methods* 2017, **14:**975-978.

30. Risso D, Schwartz K, Sherlock G, Dudoit S: **GC-content normalization for RNA-Seq data.** *BMC Bioinformatics* 2011, **12:**480.
